# Supplementary material for: Content-rich biological network constructed by mining PubMed abstracts
Source: BMC Bioinformatics. 2004 Oct 8;5:147. doi: 10.1186/1471-2105-5-147 (PMC528731; doi:10.1186/1471-2105-5-147)
Supplement: Additional File 2 — The original results of the above study (non-essential files are deleted to keep the file size under the limit set by BMC bioinformatics). [file 1471-2105-5-147-S2.bz2 › chilibotAdditionalFile2/dip10/138ID7739537E494/html/TFDP1_E2F2.html]

 


 **TFDP1** and **E2F2** 
  
Found 26 abstracts in PubMed, retrieved 10.  
 

 What does Google say? 
 PDF only 
| .edu only 

---

**Interactive relationship** (e.g. stimulation, inhibition, etc)

**Non-interactive relationship** (e.g. studied together, co-existance, homology, etc.)

- For both E2F 1 and E2F 2  [ **E2F2** ] , but not E2F 3 or E2F 5 DP1  [ **TFDP1** ] , cell cycle re entry was associated with almost quantitative cell death.  Ref: 9780003 Oncogene, 1998
- Severe deregulation of G1 S transition was also indicated by the dramatic up regulation, particularly in the HCCs, of pRb free E2F1 DP1  [ **TFDP1** ]  and  **E2F2**  DP1  [ **TFDP1** ]  transcription factor heterodimers, as assessed by immunoprecipitation and immunohistochemistry.  Ref: 9426068 Cancer Res, 1998
- No differences were observed between RA sensitive and RA resistant ovarian carcinoma cells in the levels of expression of many cell cycle genes including cyclin A, B and E, cdk and 6, E2F 1, E2F 2  [ **E2F2** ] , E2F 3, E2F 4, E2F 5, DP 1  [ **TFDP1** ]  and DP 2.  Ref: 11753676 Oncogene, 2001
